# Extreme Events Contributing to Tipping Elements and Tipping Points

**DOI:** 10.1007/s10712-024-09863-7

**Published:** 2024-11-16

**Authors:** A. Romanou, G. C. Hegerl, S. I. Seneviratne, B. Abis, A. Bastos, A. Conversi, A. Landolfi, H. Kim, P. E. Lerner, J. Mekus, B. L. Otto-Bliesner, F. S. R. Pausata, I. Pinto, L. Suarez-Guiterrez

**Affiliations:** 1 NASA Goddard Institute for Space Studies, and Department of Applied Physics and Applied Mathematics, Columbia University, New York, NY 10025 USA; 2 School of GeoSciences, University of Edinburgh, Edinburgh, EH8 9XP UK; 3 Institute for Atmospheric and Climate Science, ETH Zurich, 8006 Zurich, Switzerland; 4 Starion Group, 00044 Frascati, Italy; 5 Institute for Earth System Science and Remote Sensing, Leipzig University, 04013 Leipzig, Germany; 6 Max Planck Institute for Biogeochemistry, 07745 Jena, Germany; 7 National Research Council of Italy, CNR ‐ ISMAR ‐ Lerici, Forte Santa Teresa, Loc. Pozzuolo, 19032 Lerici, SP Italy; 8 National Research Council of Italy, CNR ‑ ISMAR ‑ Rome, 00133 Rome, Italy; 9 Moon Soul Graduate School of Future Strategy, Korea Advanced Institute of Science and Technology, Daejeon, 305-701 Republic of Korea; 10 Columbia University, New York, NY 10027 USA; 11 Autonomic Integra, New York, NY 10025 USA; 12 Climate and Global Dynamics Laboratory, NSF National Center for Atmospheric Research, Boulder, CO 80307-3000 USA; 13 Department of Earth and Atmospheric Sciences, Centre ESCER (Etude Et Simulation du Climat À L’Echelle Regionale) and GEOTOP (Research Center on the Dynamics of the Earth System), University of Quebec in Montréal, Montréal (Québec), H3C 3P8 Canada; 14 Royal Netherlands Meteorological Institute (KNMI), NL-3731 GA De Bilt, The Netherlands; 15 Institut Pierre-Simon Laplace, CNRS, 75252 Paris Cedex 05, France

**Keywords:** Extreme events, Tipping elements, Tipping points, Compound extreme events, Climate change, Climate risk

## Abstract

This review article provides a synthesis and perspective on how weather and climate extreme events can play a role in influencing tipping elements and triggering tipping points in the Earth System. An example of a potential critical global tipping point, induced by climate extremes in an increasingly warmer climate, is Amazon rainforest dieback that could be driven by regional increases in droughts and exacerbated by fires, in addition to deforestation. A tipping element associated with the boreal forest might also be vulnerable to heat, drought and fire. An oceanic example is the potential collapse of the Atlantic meridional overturning circulation due to extreme variability in freshwater inputs, while marine heatwaves and high acidity extremes can lead to coral reef collapse. Extreme heat events may furthermore play an important role in ice sheet, glacier and permafrost stability. Regional severe extreme events could also lead to tipping in ecosystems, as well as in human systems, in response to climate drivers. However, substantial scientific uncertainty remains on mechanistic links between extreme events and tipping points. Earth observations are of high relevance to evaluate and constrain those links between extreme events and tipping elements, by determining conditions leading to delayed recovery with a potential for tipping in the atmosphere, on land, in vegetation, and in the ocean. In the subsurface ocean, there is a lack of consistent, synoptic and high frequency observations of changes in both ocean physics and biogeochemistry. This review article shows the importance of considering the interface between extreme events and tipping points, two topics usually addressed in isolation, and the need for continued monitoring to observe early warning signs and to evaluate Earth system response to extreme events as well as improving model skill in simulating extremes, compound extremes and tipping elements.

**Article Highlights**

- Drought, heat, and other climate extreme events can trigger land tipping points
- Marine heatwaves, high acidity and low oxygen extremes and possibly intrinsic variability can trigger ocean tipping points
- Close monitoring, at high frequencies, including from space, is required to assess occurrence, response and resilience to extremes including as early warning signs

## Introduction

### Research Context and Scope of Article

**Fig. 1:**
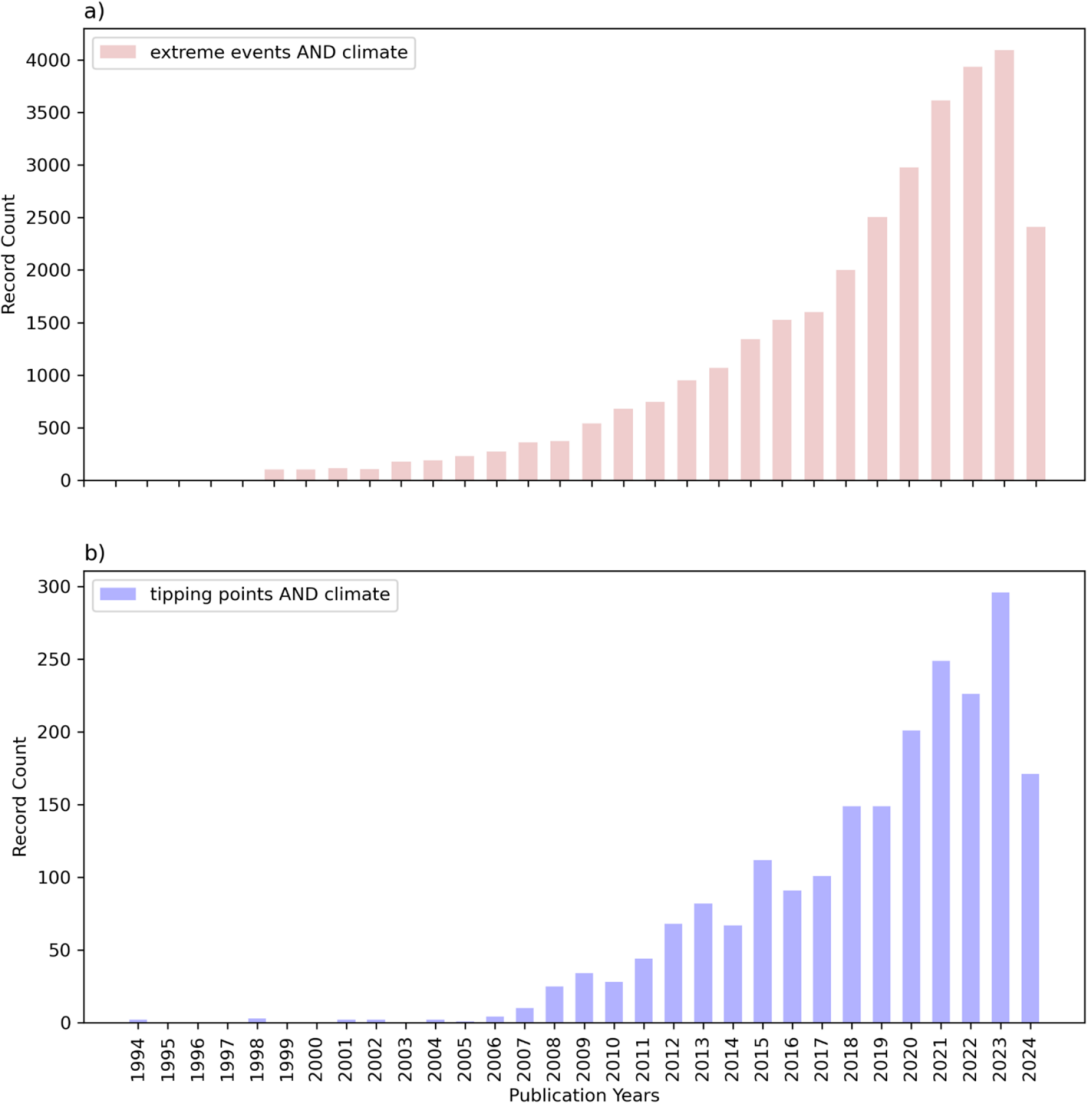
Number of publications in Web of Science Core Collection on the topic: **a** “Extreme events AND climate”; and Number of publications on the topic: **b** “Tipping points AND climate” (search: 23.07.2024)

In recent years, climate research on both extreme events and tipping points has steadily increased to respectively become (Fig. 1):

- *(Climate extreme events)* a mainstream research field addressed in several thousands of articles per year. This topic has been highlighted in the cross-Working Group Special report on Extremes (IPCC 2012), with ocean extremes discussed in the special report on ocean and cryosphere (SROCC 2019; Collins et al. 2019). More recently, extreme events were summarized in a full chapter of the latest 6th Assessment Report of the Intergovernmental Panel on Climate Change (IPCC 2021; Seneviratne et al. 2021).
- *(Tipping points)*  a research topic of growing importance in climate science which was addressed in several key articles in recent years (e.g., Lenton et al. 2008, 2023; Armstrong McKay et al. 2022; Wang et al. 2023a, b). The increasing possibility of crossing of tipping points is highlighted as a key climate risk, for example, in the World Climate Research programme lighthouse activity ‘safe landing climates’ (https://www.wcrp-climate.org/safe-landing-climates; accessed 7/2024)

Despite the prominence of both research topics, to our knowledge the links between extreme events and tipping points have not been explicitly addressed so far. In this review article, we highlight a) how extreme events in both the land and ocean systems can affect global and regional tipping points, b) the links between several types of extreme events in the context of tipping point research, c) the potential for monitoring changes in extreme events and tipping points, in particular through Earth Observations (EO) to provide early warning, and d) further avenues for research at the interface between climate extremes and tipping point research.

### Definitions

Following the terminology of Lenton et al. (2008), we refer to a **tipping point** as a critical threshold beyond which a system reorganizes, often abruptly and/or irreversibly (see Chen et al. 2021), or in other words, the point at which a small perturbation can yield large changes (“little things can make a big difference”, Gladwell (2000)). However, we do not limit our assessment to global Earth System tipping points (e.g., Armstrong McKay et al. 2022) but we also consider regional tipping points and critical thresholds in the Earth System, as well as tipping points and critical thresholds associated with society or ecosystem impacts (e.g., breadbasket failures, regional forest dieback) resulting from human-induced climate change.

Earth System **tipping elements** are at a minimum subcontinental-scale components of the Earth System that could potentially undergo state shifts triggered at some level of climate forcing by modest levels of additional forcing that exceed critical thresholds or **tipping points** (Armstrong McKay et al. 2022).

Once tipping points have been crossed, changes are not easily reversible, at least not under the same forcing conditions that triggered them, or they take significantly longer to recover than the time it took to reach them (Lenton et al. 2023).

Extreme events belong to the most visible and impactful consequences of climate change (Seneviratne et al. 2021). Here, we discuss the link between such tipping points/ and tipping elements and extreme events, including compound extreme events.

Extreme weather and climate events are defined as follows in the 6th Assessment Report of the Intergovernmental Panel on Climate Change (IPCC; see also Seneviratne et al. 2021): An **extreme weather event** is ‘an event that is rare at a particular place and time of year’, and an **extreme climate event** is ‘a pattern of extreme weather that persists for some time, such as a season’. **Rare events** are typically in the extremely low or high range of a given variable, and thus extreme events are often also defined in terms of their probability of occurrence, such as exceeding the 90th or 99th percentile of temperature of a climatological baseline, e.g., a preindustrial, mid-twentieth century, or late-twentieth century baseline, or a moving-mean baseline, but it depends on the study.

In the context of tipping points, compound events, a subcategory of extreme events, can be of particularly high relevance. **Compound weather and climate events** describe combinations of multiple climate drivers and/or hazards that contribute to societal or environmental risk (Zscheischler et al. 2018; Bustamante et al 2023). Examples of compound events that may lead to long-lasting impacts include drought with heat or fire, multiple storm hits (these are temporally compound), and extremes of ocean temperature occurring together with high acidity. Extreme events, particularly compound events, can also lead to cascading impacts and chains of subsequent events (e.g., AghaKouchak et al. 2020). As compound weather and climate events may occur more frequently in many regions, especially in relation to high emission scenarios (Ridder et al 2022), their potential triggering of tipping points needs to be monitored.

**Fig. 2:**
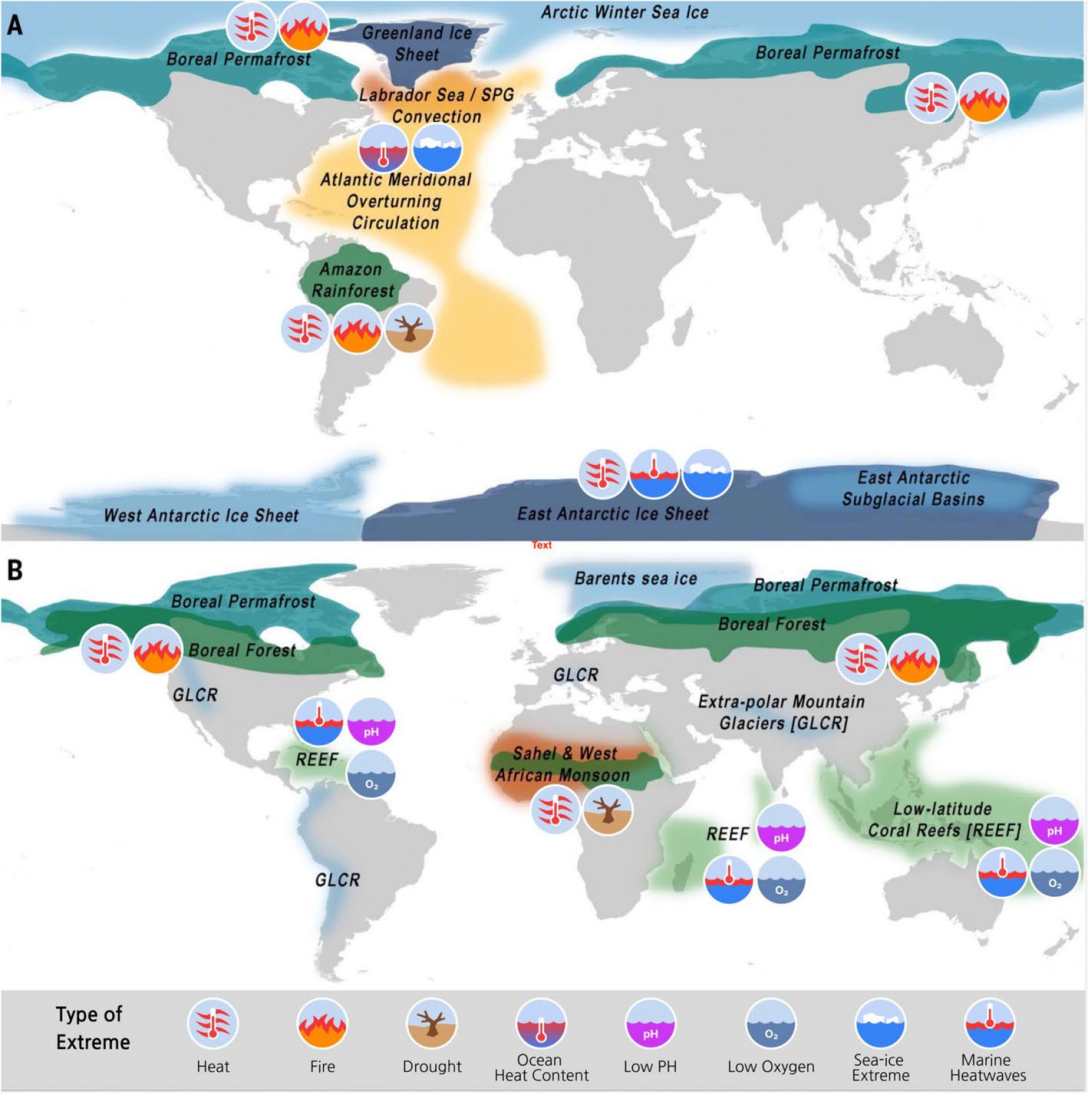
Schematic of extremes and compound extremes on land and ocean and their link to tipping elements of global (**A**) and of regional (**B**) impact, drawing on Fig. 1 from Armstrong McKay et al (2022). Symbols indicate links to extreme events. For land: Boreal forest responds to drought, fire, heat; Boreal permafrost to heat; Amazon rainforest to drought and fire. Mountain glaciers (GLCR) respond to heat. For ocean: AMOC responds to marine heatwaves and low salinity extremes, coral reefs respond to marine heatwaves, high acidity, and low oxygen concentration extremes

Figure 2 displays global and regional tipping elements identified in the study of Armstrong McKay (2022) and their links to some weather and climate extreme events. We note that not all tipping points and elements are necessarily affected by extreme events, and that even for the tipping elements which are influenced by extreme events, other non-extreme conditions and non-climatic processes can play a role as well. We focus both on changes with global and large-scale ramifications, and also refer to events where tipping occurs on the interface between ecosystems and society.

**Fig. 3:**
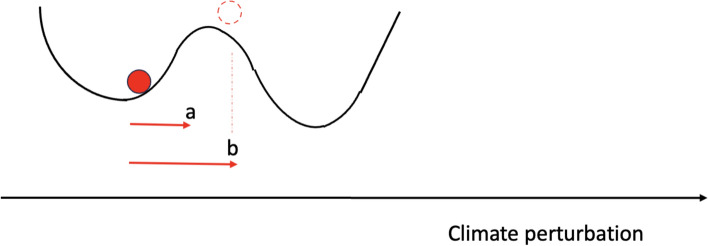
Links between extreme events (arrows) and the crossing of a critical threshold associated with a tipping point (dashed line circle). Compared to a standard perturbation (“**a**”), an extreme event (“**b**”) can push the system sufficiently away from its base state (filled circle) to induce a tipping point. The extreme event could also be a compound event, or a sequence of multiple events (temporally compound)

Figure 3 illustrates how extreme events can push a given system beyond a critical threshold determining a tipping point, related to the concept of a “noise-induced tipping point” (Ashwin et al. 2012; Boers et al. 2022). Here we focus on cases where the amplitude of a severe extreme event pushes the system further toward the alternative state than less extreme variability does. For example, noise-induced transitions in AMOC have been explored in several studies (e.g., Castellana and Dijkstra 2020). Long-term impacts of some extremes, such as vegetation loss to fire or drought, and degradation may also lead to a persistent alteration of forest properties and thus diminish the viability of the previous state (e.g., Saatchi et al. 2021).

In this article, we review the literature on weather and climate extreme events, including compound events, which might be relevant in the context of tipping points or tipping elements. We discuss the extent to which extreme events can:

- Contribute to reported global Earth System tipping points, such as Amazon rainforest loss, the Atlantic Meridional Overturning Circulation (AMOC) shutdown, or loss of major ice sheets (see Armstrong McKay et al. 2022; Wang et al. 2023a, b).
- Contribute to regional climate tipping points of the Earth system, both those highlighted in review papers (e.g., Boreal forest change, see Wang et al. 2023a, b) and other cases where extreme events may lead to long-lasting or irreversible changes, such as long-lasting vegetation change.
- Contribute to global and regional society and ecosystem tipping points, such as migration in the dustbowl region in response to drought, degradation of ecosystem services, food insecurity and other economic pressures due to coral and fisheries collapse and loss of regional biodiversity; humanitarian crises in response to extremes.
- We also identify evidence of processes that increase Earth System resilience, i.e. processes which lead to increased carbon uptake following some extremes.

This paper is organized as follows: We first show from the perspective of both observations and process studies how extreme and compound events on land (Sect. 2) and in the ocean (Sect. 3) contribute to tipping points. This is followed by a discussion of the state of understanding based on paleoclimate data (Sect. 4), observations and climate modeling (Sect. 5), and finally recommendations and opportunities to use Earth observations (EO) for early warning of extreme events that can lead to tipping.

## Land Extreme Events from a Tipping Point Perspective

### Overview of Link between Extreme Events and Tipping in Land Systems

*Global climate tipping elements* arising from land systems include the Amazon rainforest, the boreal permafrost, and major glaciers and ice sheets (Armstrong-McKay et al. 2022, see also new combined schematic Fig. 2), and arguably all of these are sensitive to large-scale extreme events, as highlighted hereafter. In addition, changes in the boreal forest have been identified as a tipping element and are also sensitive to extreme events. Finally, extreme events could also trigger impact-related tipping points in the land systems both on a regional and global scale.

#### Amazon Rainforest (Global Tipping Element)

The Amazon rainforest shows well-documented vulnerability to extremes, particularly increasing drought (combined with extreme temperatures) under increasing global warming (Seneviratne et al. 2021), and enhanced fire activity, which is amplified by human disturbance (Hubau et al. 2020; Silva Junior et al. 2021; Rosan et al. 2022; Lapola et al. 2023). The combination of these changes could lead to irreversible degradation of the forest, and with it its tipping toward a degraded forest or savanna state (see review in Armstrong McKay et al. 2022; Wang et al. 2023a, b; Nobre et al. 2016; see Fig. 2).

Due to its role in water recycling, Amazon rainforest losses are expected to be self-amplifying (Zemp et al. 2017) and to have global ramifications (Lenton et al. 2008), such as disruption of water transport to important agricultural regions of the world, with irreversible effects for at least decades. The main drivers of Amazon forest loss in models and observations are deforestation and the effects of climate change, such as increased soil moisture drought associated both with increased evaporation and decreased precipitation as well as the combination of dry and hot conditions leading to a stronger incidence of fire. Analyses have already identified the Amazon as a sensitive system in a degraded state (Saatchi et al. 2021; Boulton et al. 2022; Lapola et al. 2023). Armstrong McKay et al. (2022) provide a best estimate that this tipping point may be triggered around 3 °C of global mean warming since the preindustrial period (with minimum value of 2 °C).

However, uncertainties about the future response of the Amazon forest to climate extremes are substantial, especially about the impact of their interactions. For example, recent research suggests that deforestation impacts water recycling and can increase the possibility of forest loss (e.g., Bochow et al. 2023); but also impacts the precipitation regime in the dry season in complex ways, which might lead to opposing effects (Khanna et al. 2017). The realism of processes linked to the land–atmosphere coupling under drought in climate models is also uncertain. For example, soil moisture and rainfall may be coupled too strongly in low resolution models (Lee and Hohenegger 2024). There is also high uncertainty about the ability of state-of-the-art Earth System Models (ESMs) to simulate forest resilience to climate change. Some studies indicate that ESMs underestimate the sensitivity of ecosystem carbon uptake to drought variability under the current climate (Humphrey et al. 2018), and others report an observed increase in this sensitivity in the recent decades (Liu et al. 2023a, b), thus challenging the assessment of the exact conditions under which the Amazon rainforest dieback as global tipping point could be triggered. Also, fires at the edges of humid forests could play an important role in this context (Silva Junior et al. 2020). Furthermore, Earth System models lack the representation of processes that could confer increased resilience of the Amazon rainforest to climate extremes, such as vegetation demographics and biodiversity which can contribute to buffer impacts and enhancing ecosystem resilience (Sakschewski et al. 2016; Fisher et al. 2018). On the other hand, they generally also do not represent drought-induced forest mortality adequately, and there is evidence that they may not be able to represent fires as large as some recently observed, at least not in Australia (Sanderson and Fisher 2020). Therefore, while some processes not represented in Earth System Models could imply a higher resilience, the balance of evidence suggests that the feedbacks triggering this tipping point may be underestimated in current models and thus this tipping element could be triggered at a lower level of global warming than estimated in Armstrong McKay et al. (2022), which would have substantial policy implications. More research is necessary to clarify this point.

#### Boreal Permafrost (Global Tipping Element)

Boreal permafrost could thaw under rising temperatures, affecting the natural and built environments and water cycle balance of the region and further releasing currently trapped methane into the atmosphere (Smith et al. 2022; see companion paper by Brovkin et al., 2024). This is first a regional tipping element due to its importance to the regional water cycle, and second permafrost thaw can also have global implications due to possible carbon and methane release. Permafrost change has been linked to vegetation changes and extreme events, such as extreme heat, wildfires, and precipitation shifts (see Wang et al. 2023a, b).

#### Glaciers and Ice Sheets (Global Tipping Element)

As temperatures continue to rise, glaciers and ice sheets will also continue to melt, leading to potentially irreversible ice mass loss (Hanna et al. 2020) and, in turn, to impacts ranging from irreversible sea level rise (Frederikse et al. 2020) to changes to the Earth's albedo that could further exacerbate both warming and ice melt (Box et al. 2012). The melting of large ice sheets is interlinked with complex processes on the interface between ocean and ice creating substantial uncertainty in the dynamics and future evolution of major ice sheets. Furthermore, ice-sheet melting can be exacerbated by hot spells, as exemplified by the 2010 record melting in the Greenland Ice Sheet (Tedesco et al. 2011). In 2010, many locations in Greenland experienced record-breaking temperatures in winter, spring, summer and the year as a whole. The combination of abnormally warm conditions both in the cold and in the warm seasons lead to a melting period 50 days longer in 2010 compared to the 1979–2009 average, which started exceptionally early and ended exceptionally late (Tedesco et al. 2011).

#### Boreal Forest

Beyond the above-mentioned global tipping elements, another tipping element in the land systems includes shifts (Northward expansion and Southward retreat) in the Boreal forest. It is listed as a regional tipping element in Fig. 2, although it may have global consequences on Earth's albedo and is classified as a global tipping element in another recent review paper (Wang et al. 2023a, b). Warming, enhanced by Arctic amplification, leads to growing season shrub encroachment in the high Arctic (e.g., Myers-Smith et al. 2015), causing an expansion of tree cover into currently treeless tundra ecosystems, at rates of up to 4 km per decade (Dial et al. 2022). However, the Southern parts of the boreal forest are in retreat attributed to fire and increased logging (Rotbarth et al. 2023; see also Beck et al. 2011; Carpino et al. 2018; Wang et al. 2023a, b; and references therein). Empirical evidence suggests that disturbances are responsible for switches between alternative states rather than gradual changes (Scheffer et al. 2012; Abis and Brovkin 2017; Lenton et al. 2023), for instance due to shift from evergreen to deciduous species (Mack et al. 2021; Massey et al. 2023). In many events (see discussion below), extreme heat, fire and ecological changes are tightly linked in high latitudes, given that vegetation and climate can feed back on each other (Bastos et al. 2023). For example, compositional changes due to more frequent fires in Alaska have been reported to result in biomass gains (Mack et al. 2021) due to feedbacks with nutrients and other ecological processes. Boreal forest change can be globally important because it has the potential to influence warming by changing albedo (Massey et al. 2023), yet at present the impacts of forest changes are uncertain, and their potential to lead to tipping are underexplored.

#### Further Land-Related Tipping Elements, Including in Ecosystems and Society

Further tipping elements are linked to the monsoon systems, particularly the Sahel and West African monsoon system (see schematic in Fig. 2; Armstrong McKay et al 2022), which may not be per se irreversible (Wang et al. 2023a, b), but can have huge societal consequences. However, the mechanisms are complex, such as changes in the onset and retreat of monsoons and thus not clearly linked to individual extreme events. Other changes related to tipping in the literature include long-term change in heat/moisture climate due to soil-moisture changes in heatwaves, such as in Central Europe (Seneviratne et al. 2006) and in a case in central East Asia (Zhang et al. 2020).

Furthermore, extreme events may lead to cascading effects and socioeconomic tipping in the human system, for example through heat and drought-induced failure of breadbasket regions, and long-lasting impacts or transitions in societies affected by them; thus, leading to global and regional tipping elements affecting society and ecosystems.

In the following, we discuss the mechanisms by which specific types of extreme events may lead to long-lasting changes, threshold behavior and with it, tipping points. We focus on drought and heat given the larger body of literature focusing on these events, but long-lasting impacts are not limited to them. For example, high-impact compound events can also arise from severe storm hits, particularly in sequence. An example is multiple or a single large tropical cyclone landfall, which creates potential for severe and long-lasting flooding, and impacts the viability of infrastructure. Multiple storm hits can lead to very slow recovery times and impact habitability, with a negative impact on community resilience (e.g., Binte and Hasan 2023).

### Drought-Heat-Fire Events and Their Potential Link to Tipping

Headline messages from the recent Intergovernmental Report on Climate Change (IPCC 2021) indicate that extreme high-temperature events that used to recur every 50 years in a preindustrial climate, now are likely to occur about every decade, and will potentially increase to every 3–4 years at 2 °C of global warming, and such heatwaves are also expected to be more intense. The frequency of drought has also already increased with a previous 10-year event expected to be more than twice as frequent, and more intense, in a 2 °C warmer world (Seneviratne et al. 2021).

As discussed above, both Amazon rainforest dieback and boreal forest changes have the potential to interact with severe and large-scale extreme events. However, here we argue that other regions might also be sensitive to long-lasting, near-term irreversible impacts from large-scale severe events, which can lead to long-term consequences, such as vegetation loss or change (e.g., Seidl and Turner 2022). We discuss mechanisms by which extremes could create long-lasting transitions in the Earth system. For example, an increase in compound hot-dry conditions is also a key characteristic of projected climate change in the Mediterranean region (Seneviratne et al. 2021), where it could potentially lead to a marked loss of local vegetation beyond 2 °C of global warming (Guiot and Cramer 2016).

Co-occurring or ‘compound’ drought and heat events amplify each other, both in terms of intensity and in terms of impacts (Seneviratne et al. 2010; Allen et al. 2015; Zscheischler and Seneviratne 2017; Zscheischler et al. 2018). In particular, under soil-moisture limitation and increased atmospheric aridity, vegetation productivity and stomatal conductance decline (Fu et al. 2022), reducing surface cooling, with resulting increased heat enhancing evaporative demand and creating a positive feedback (e.g., Seneviratne et al. 2021). Where physiological or ecological thresholds are crossed (e.g., Lawrence et al. 2022; Allen et al. 2015; Hartmann et al. 2022), dieback can occur; increased wildfires result in carbon stocks that are rapidly released into the atmosphere, followed by increased carbon sequestration as vegetation regrows (Byrne et al. 2021; Qin et al. 2022). Furthermore, after severe compound drought and heat stress, vegetation recovery can be delayed, for example due to reduced growth or non-reversible damages (Ruehr et al. 2019), and potential impacts may be worsened by increasingly frequent successive or multi-year extremes (Suarez-Gutierrez et al. 2023). This may lead to legacy effects (Ruehr et al. 2019; Kannenberg et al. 2019; Yu et al. 2022a, b) that increase vulnerability to further compound drought and heat stress. If subsequent events occur before complete vegetation recovery, they can lead to larger impacts, for example as occurred in the 2018–2019 successive extreme hot and dry summers in central Europe (Bastos et al. 2021a, b). Widespread tree mortality in the aftermath of these events (Hlàsny et al. 2021) have turned the forests in the Czech Republic into a carbon source (EUCRA 2024). Thus, crossing a threshold for the coupled vegetation/climate system, or cumulative effects of repeated extremes, can create a regional ‘tipping point’ where a small perturbation can cause a rapid, nonlinear change into a different state.

Fire is endemic to some forest or wooded ecosystems, which are in fact often dependent on fire (e.g., Bowman et al. 2009), but these are adapted to their corresponding fire disturbance regime (return period, magnitude, severity; Turner 2010). Changes in fire regimes such as increased fire return frequencies and fire intensity, or an expansion of fires into less frequently burnt areas, can lead to vegetation loss, productivity changes, or shifts to a different vegetation state altogether (Turner and Seidl 2023). For instance, a decrease in coniferous forests, potentially replaced by early successional broadleaved tree species, or a decrease in fire-resisting tree species (Anoszko et al. 2022; Liu et al. 2022; Abis and Brovkin 2019). Wildfires can also affect the regional water cycle (e.g., Zemp et al. 2017).

**Fig. 4:**
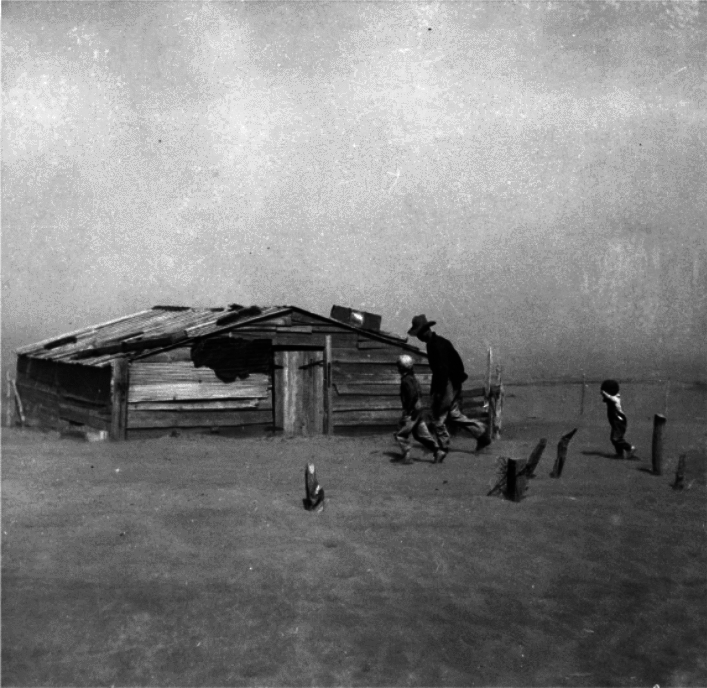
A farmer and his sons were caught in a dust storm in Cimarron County, Oklahoma, April 1936. NPx 66-174(32) from Forward with Roosevelt; F.D. Roosevelt presidential library and museum

The following two examples highlight the potential for extreme events to lead to severe, long-lasting and sometimes surprising consequences:

The first such example is the extreme US ‘Dust Bowl’ drought (Fig. 4), and resulting severe land surface changes. It was initiated by long-term (Schubert et al. 2004) but not unique drought (Cook et al. 2022a, b). The ‘Dust Bowl droughts’ contributed to extreme heat (Cowan et al. 2017), crop failure and dust storms in some cases reaching the US East Coast, which in turn amplified drought and heat (Cook et al. 2008) with serious consequences for human health and migration (Worster et al., 1979; see review in Hegerl et al. 2018). Some ESMs simulate dust resulting from vegetation loss (see Cowan et al. 2020a,b). This event was caused by a combination of extreme events and societal factors (rapid transition of the great plains to agriculture, which was vulnerable to drought) and led to a tipping point in the human system, but also to very long-lasting (and in some locations still not reversed) changes in the land surface, hence breached regional tipping points.

A second example that illustrates potential for severe and possibly irreversible change is the occurrence of recent extreme fire seasons in high latitudes, including in the summers of 2019–2021 in Siberia (Scholten et al. 2022), where many strong fire events in recent decades were preceded by severe heat (Hegedűs et al. 2024). Recent fires and droughts in Siberia have decreased its carbon sink (Fan et al. 2023). The 2020 Siberian heat wave is considered to have been almost impossible without human influence on climate (Ciavarella et al. 2021) and led to particularly strong fire activity. However, the timing of the events is also important, for example the 2020 Siberian heat wave led to an increase in spring vegetation productivity, which offset later losses in summer and fall (Kwon et al. 2021). Northern Canada has seen an intense fire season in 2023, with evacuation of the town of Yellowknife and smoke emissions from the fires affecting air quality across a broad swath of North America (Wang et al. 2023a, b). These fires also released a record amount of carbon into the atmosphere, with possible implications for carbon budgets (Zheng et al. 2023), although recovery needs to be assessed. Nevertheless, the increased rates of change and unprecedented magnitude of many recent extremes highlight risk and change in the boreal forest.

Several other recent extreme events caused severe and slow-to-reverse impacts regionally, some with global ramifications that illustrate the potential for even more severe extreme events to cause tipping, particularly in ecosystems and human systems. For instance, the 2003 European heat wave was a record-shattering extreme event (Schär et al. 2004) that led to tens of thousands of excess deaths, severe drought stress on vegetation, loss of agricultural production, and multiple other consequences. The 2021 Pacific Northwest Heat wave saw record-shattering heat (White et al. 2023)—where the highest temperature measured ever above 50° of latitude (49.6 °C) occurred—an intense fire season, and vast agricultural and ecological losses, including the deaths of more than 10 million marine animals such as barnacles and sea stars due to the co-occurrence of low tide in South facing shores with peak daily temperatures (White et al. 2023). Severe vegetation impacts resulted from the hot and dry European summer of 2019. The Horn of Africa has recently experienced severe drought exacerbated by heat (Kimutai et al. 2023) with devastating consequences for populations living there. In the Southern Hemisphere, recent Australian summers have broken records for heat and wildfires and have been linked to greenhouse gas increases (van Oldenborgh et al. 2021). They have also been a driver of mass fatalities of some arboreal mammals by breaching their temperature tolerance (Ratnayake et al. 2019), leading for example to the death of more than 70.000 Australian flying foxes in the 2019–2020 summer, triggered first by drought-caused starvation and pup abandonment, second by heat stress related die-offs, and third by wildfires (Mo et al. 2021).

**Fig. 5:**
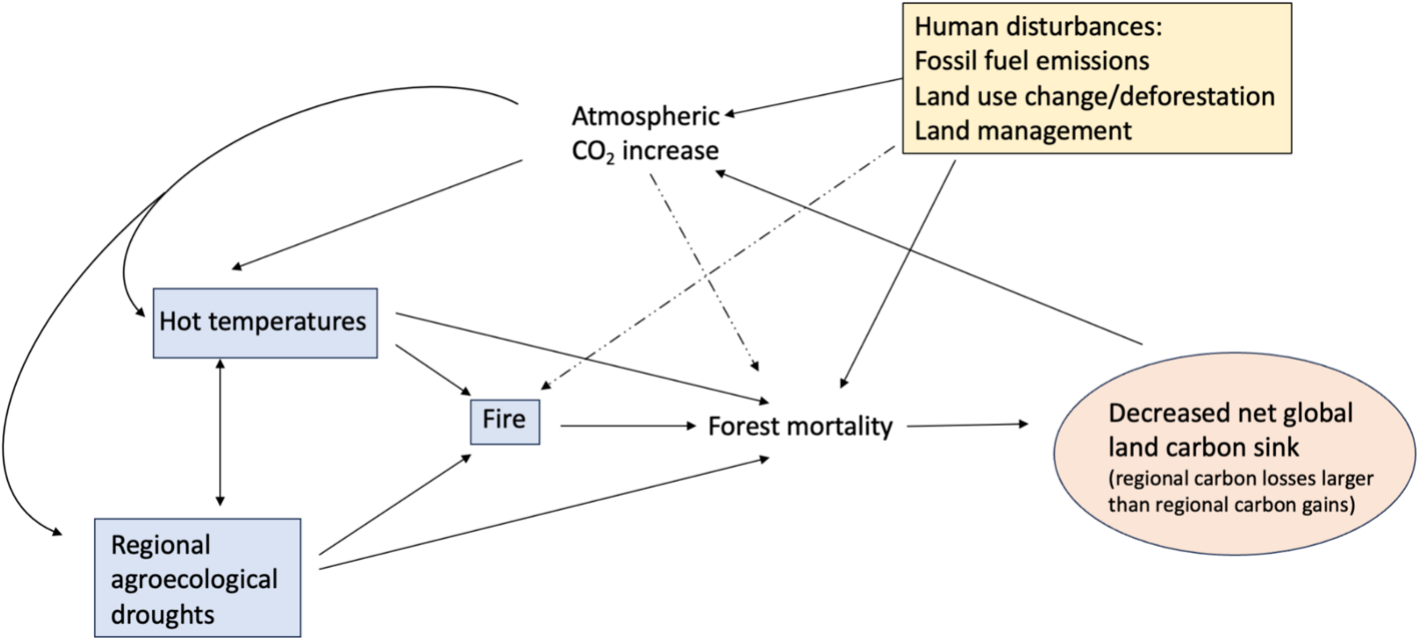
Links between extreme events, human disturbances, and a potential carbon sink-related tipping point related to spatially and temporally compounding effects of unprecedented impacts of heatwaves, droughts and associated fire and forest mortality discussed in Sect. 2.2. Extremes are indicated by blue rectangles, the tipping element (decreased global land sink) with the orange oval, and the human disturbances with the yellow rectangle. Solid arrows indicate an amplifying mechanism, while dash-dotted lines indicate relationships that can either amplify or dampen the response. Regarding the global land carbon sink tipping point, it corresponds to a globally aggregated impact, and does not exclude the presence of regional carbon gains for some events (e.g., in Scandinavia during the 2018 heat-drought event). On average, and despite these possible regional exceptions, heat-drought events are typically associated with regional carbon losses that can take several years to recover and might be irreversible if extreme events recur faster than recovery times. Note that the figure highlights CO_2_, but warming due to other greenhouse gases contributes as well to climate change

In summary, compound heat and drought extremes events can enhance each other, and can trigger degradation of vegetation, agricultural, animal and human loss, and severe wildfires. These, in turn, can cause cascading impacts with vegetation changes potentially feeding back on climate (see hereafter and Fig. 5). Several factors make the occurrence of local or regional tipping points more likely: the increase in compound hot-dry events across the world; associated changes in disturbance regimes (fires, insect outbreaks, drought-induced mortality); and the fact that carbon losses are fast, but recovery is slow and potentially hampered by climate change. When aggregated spatially and in time, the cumulative effects of such impacts, even beyond the Amazon and boreal regions, are likely to reduce the efficiency of the global land carbon sink beyond what is currently projected (Bustamante et al. 2023; see Humphrey et al. 2018, Green et al. 2019 for examples), thereby further amplifying warming. There is currently medium confidence that such changes can result in positive carbon-climate feedback from climate extreme driven disturbances (Canadell et al. 2021).

We also note that tipping point or threshold behavior may arise in the response by society, such as out-migration (for an example see the dust bowl case study above), which may also occur in response to present day extreme events threatening livelihoods.

### Concurrent/Teleconnected Extremes in Breadbasket Regions

Hot and dry extreme events also have the potential to risk severe consequences on society through their impact on crops. Heat and drought may be especially damaging when extreme conditions occur during the much more vulnerable sprouting and flowering periods in the spring (Senapati et al. 2021; Luo et al. 2023). Where heat and drought events occur in agriculturally important ‘breadbasket’ regions, they can reduce harvests and threaten global food security (Kornhuber et al. 2020). Recent studies showed that with advancing global warming, the area affected by extreme events increases substantially (Vogel et al. 2019; Batibeniz et al. 2023), including in breadbasket regions (Gaupp et al. 2019; Seneviratne et al. 2021; Biess et al. 2024), and several breadbasket regions may be affected by extremes simultaneously more frequently (Raymond et al. 2022). Recent research also indicates that extreme heat in one region could be dynamically linked to heat in other regions, some of these breadbasket regions (Meehl et al. 2022), highlighting the risk of spatially compound heat events affecting more than one breadbasket region.

Concurrent failure in breadbasket regions can result in increased food prices and limited access to resources, affecting both the population and humanitarian organizations trying to procure and distribute aid (IPCC 2019; Mbow et al. 2019; Kornhuber et al. 2022). Food insecurity associated with pre-existing vulnerabilities can affect human migration patterns. Also, coordinating and delivering humanitarian aid in the aftermath of spatially compounded events within the same country can pose significant logistical challenges. An example of this complexity is evident in the case of Tropical Cyclone Freddy, which impacted Mozambique during two distinct periods: from 6–24 February and 2–14 March 2023 (OCHA 2023).

To our knowledge, literature assessing potential regional or global societal tipping elements associated with concurrent extremes, such as breadbasket failures, is not yet available, and it is an area that should be more investigated in near future. This is in particular the case given the identified underestimation of associate impacts in current climate models (Kornhuber et al. 2022).

### Dangerous Heat and Humidity

Thresholds also exist for the human body. Under concurrent high temperatures and high ambient humidity, evaporative cooling through sweat loses efficiency and we become unable to maintain a stable body temperature, leading to potentially lethal damages (Vecellio et al. 2022; Sherwood and Ramsay 2023). While the relationship between extreme heat and human health is complex (e.g., Lo et al. 2022), recent empirical physiological research in humans finds lower humid heat thresholds than previously thought for which heat stress becomes uncompensated in young, healthy adults performing normal daily activities (Vecellio et al. 2022; Vanos et al. 2023).

Multiple indices exist for evaluating the health risk from heat stress, but the broad picture is similar between them: as global mean temperature increases beyond 1.5, 2 and 3 degrees of warming, an increasing fraction of the population will be exposed to dangerous humid heat, particularly in East Asia (Freychet et al. 2022; Suarez-Gutierrez et al. 2020). Such dangerous heat is not only linked to increased heat-related mortality and morbidity, but has also been connected to large economic losses due to decreased labor productivity (Dunne et al. 2013; García-León et al. 2021). Furthermore, assessments of extreme humid heat stress conditions may underestimate part of the risks due to the incorrect representation of relevant atmospheric and geographical effects in coarse resolution simulations, such as used in Freychet et al. (2022), underestimating humid heat in microclimates near coasts, and in cities. Lastly, as discussed above in the case of Australian flying foxes, severe heat can also cause severe impact on wildlife and with it impact on biodiversity.

The case of humid heat and impacts of extremes on food system illustrate that weather and climate extremes can lead to slow-to-reverse change or tipping in impacts, even where the extremes themselves show no such threshold behavior.

## Marine Extreme Events from Tipping Point Perspective

### Extremes, Compound Extremes and Tipping Elements in Marine Systems

The rise of atmospheric CO_2_ concentrations is a major contributor to ocean warming, ocean deoxygenation and is directly responsible for acidification (Gruber 2011). At the same time, a strengthened hydrological cycle due to warming is expected to increase the intensity and frequency of extreme weather events (see Seneviratne et al. 2021), modulating variations in evaporation and precipitation over both the ocean and the land, hence altering freshwater inputs to the ocean, which impact salinity, stratification, and consequently, circulation and oxygen supply (Fennel and Testa 2019). Modified ocean–atmosphere heat exchanges are also expected to affect wind patterns over the open ocean (Fox-Kemper, et al. 2021) while intrinsic climate variability (e.g., the El Nino-Southern Oscillation (ENSO)) which may be changing regionally (Beobide-Arsuaga et al. 2021) will also play a role in enhancing these forced trends, albeit differently in different ocean basins. All these changes carry high potential impacts for marine ecosystems and ocean biogeochemistry (e.g.: Bopp et al. 2013; Landolfi et al. 2017). As the background state changes, the likelihood of extreme conditions, that lead to global or regional tipping points with severe impacts on marine ecosystems and human services, is expected to increase as well.

Global climate tipping elements in the ocean considered in this paper (Fig. 2) are the Atlantic Meridional Overturning Circulation (AMOC), the North Atlantic subpolar gyre (SPG) mixing, and the ocean oxygen content, while regional tipping elements include low latitude coral reefs (McKay et al., 2022; Wood et al., 2024).

Extending the definitions of extremes discussed in Sect. 1.2 into the ocean realm, marine extreme events (Fig. 2) are defined when ocean properties significantly exceed the typical seasonal variations and persist for at least five consecutive days (Hobday et al. 2016) and extend for several kilometers (Frölicher et al. 2018). Under continuing global warming, marine heatwaves (MHWs—anomalous periods of elevated temperatures), low oxygen (LOX; Gruber et al. 2021), high acidity (OAX; Burger et al. 2020; 2022) and low chlorophyll (LChlX; Le Grix et al. 2021) extremes have become more frequent and some are expected to intensify in frequency, duration and areal coverage under sustained anthropogenic emissions (Gruber et al. 2021; Burger et al. 2022; Frolicher et al. 2018).

Furthermore, multiple extremes may co-occur as “compounds” in space and time, at the ocean surface (Le Grix et al. 2021; Gruber et al. 2021; Burger et al. 2022; Zscheischler et al. 2018) and/or at depth (Gruber et al. 2021; Wang et al. 2023a, b), severely threatening marine organisms and compressing habitability. For example, thermal stress during prolonged MHWs disrupts the relationship between corals and their symbiotic microalgae, causing coral ‘bleaching’, often resulting in widespread coral death (e.g., Klein et al. 2022 and references therein). Higher acidic conditions are further expected to reduce coral tolerance to heat stress (Anthony et al. 2008), leading to reduced survival of corals under intermediate and high emission scenarios (Klein et al. 2022). According to how compound events unfold in space and time, diverse typologies have been identified: (i) *preconditioned,* where a weather or climate event preconditions the impact/occurrence of the extreme; (ii) *multivariate,* when co-occurrence of multiple extremes leads to an impact; (iii) *temporally compounding*, when temporal succession of multiple extreme leads to an impact; (iv) *spatially compounding,* when extremes are occurring in multiple locations and lead to synchronized/ aggregated impact (Zscheischler et al. 2020). In the following we will use the term “compound” events to refer to multivariate, spatially and temporally compounding extremes. Preconditioned and compound extremes will be further discussed in Sect. 3.3.

**Fig. 6:**
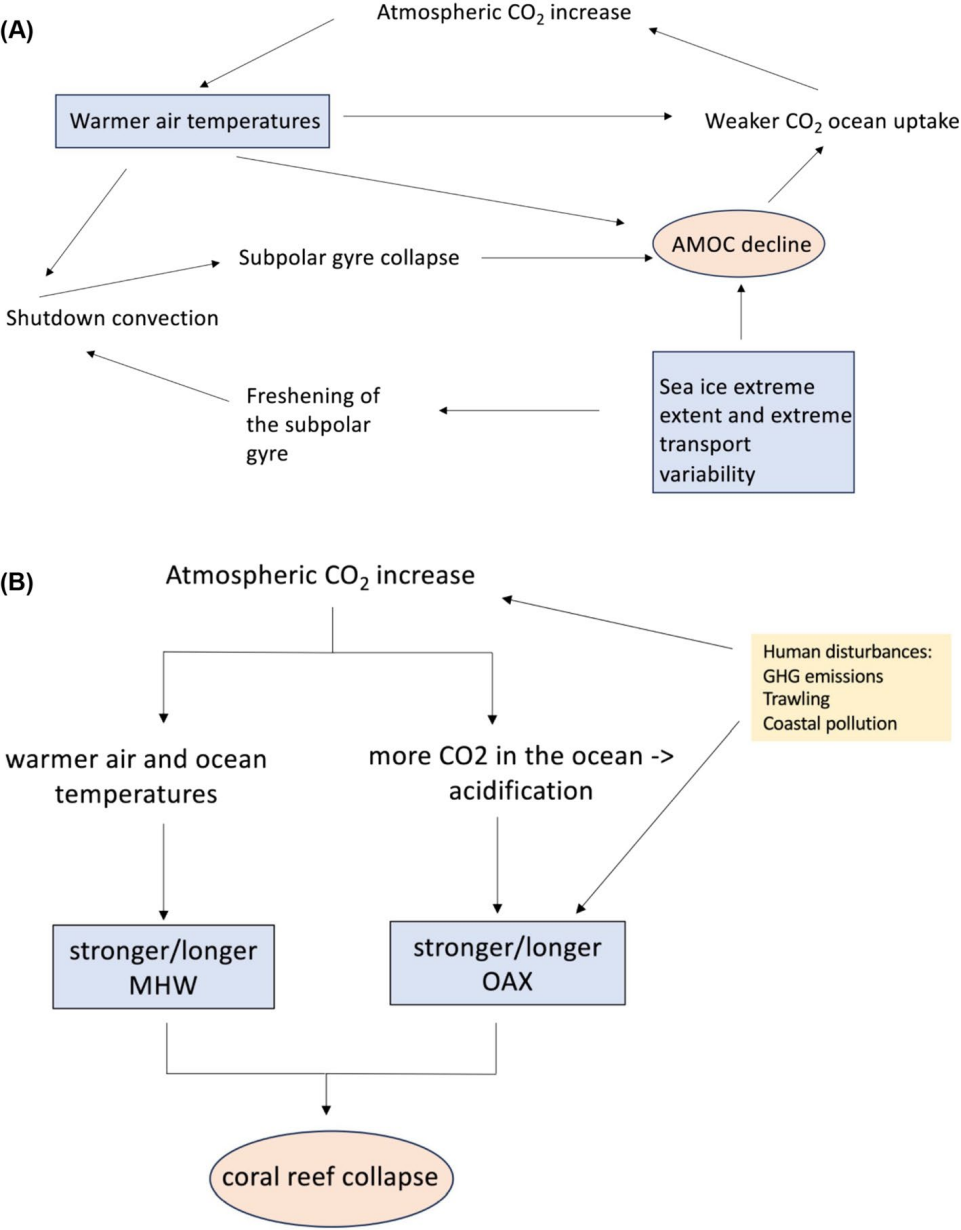
Examples of compound extremes that can lead to tipping points in the ocean. **A** Compound extreme variability of sea ice extent and sea ice transport out of the Arctic Ocean and into the subpolar gyre, leads to freshening in the SPG and causes AMOC decline (Romanou et al. 2023). **B** Compound extremes such MHWs and OAX, leading to collapse of low latitude coral reefs. Extremes are indicated by blue rectangles, the tipping elements (AMOC decline; coral reef collapse) by orange ovals and human disturbances that exacerbate marine extremes are shown in the yellow box

In addition to MHWs-OAX-LOX-LChl-primary productivity combinations of extremes, multivariate compound extremes can also coincide with extreme changes in ocean heat uptake, sea ice distribution and mass/heat/salt transports, precipitation, wind stress over the ocean surface, hurricanes, dust storms and other aerosol deposition processes, and sea level rise. Such combinations in conjunction to a changing background can lead to tipping points. For example, extremes in sea ice distribution and sea-ice transport into the subpolar gyre were shown to potentially contribute to the tipping of the AMOC under an extended SSP2-4.5 scenario in the GISS climate model (Romanou et al. 2023) through a feedback loop shown in Fig. 6A (see also discussion in Wood et al. 2024). There, increased GHGs and warming, lead to AMOC declines that triggered intense variations in sea ice and sea ice transport across the Denmark Straits and into the subpolar gyre where they melt, freshen the ocean surface, and shut down mixing and hence deep-water formation (see also Romanou et al 2023).

Combinations of extreme conditions in the ocean can have impacts that exceed the impacts of the singular events (Smith et al 2019; Lucey et al 2022; Liu et al. 2023a, b) and therefore may lead to regional tipping points. For example, acting in unison, MHW and OAX may cause tipping in coral reefs at 2 °C warming levels, particularly in the tropical oceans (Armstrong McKay et al. 2022; Klein et al. 2022; Hoegh‐Guldberg et al. 2018; Heinze et al 2021; Pearce-Kelly et al 2024; Fig. 6B).

### Role of GHG Forcing and Internal Climate Variability in Linking Extremes and Tipping Points in the Ocean

Anthropogenic forcing, i.e. increasing greenhouse gas (GHG) emissions and the ensuing global warming are the dominant drivers of the long-term changes in many extreme events, such as MHW (Frölicher et al. 2018; Oliver et al. 2018; Holbrook et al. 2019) and open ocean low oxygen extremes (Gruber et al. 2021), while CO_2_ increase drives high acidity extremes (Gruber et al. 2021; Burger et al 2020; 2022). Yet large-scale natural modes of climate variability, e.g., ENSO, NAO (North Atlantic Oscillation), PDO (Pacific Decadal Oscillation) etc. and their associated atmospheric and oceanic teleconnections, can favor or suppress the occurrence of extremes by modulating local conditions (Köhn et al. 2022; Oliver et al. 2018; Holbrook et al. 2019, 2020; Rodrigues et al. 2019; Le Grix et al. 2021). Therefore anthropogenic forcing trends as well as intrinsic climate variability might trigger and/or amplify extremes or compound extremes (Special Report on the Ocean and Cryosphere in a Changing Climate (SROCC) 2019) and thus lead to tipping points.

While the forced impacts on extremes are better understood, the role of natural climate variability is less clear. Internal climate modes regulate the likelihood of extremes due to a complex interplay of direct and indirect effects on physical, chemical, and biological processes, which are difficult to predict on short time scales. For example, changes in mixed-layer depth and upwelling strength, which are strongly modulated by ENSO, have been shown to affect the frequency of compounding MHW and LChlX events (Le Grix et al. 2021; Le Grix et al. 2022). On the contrary, the likelihood of compound MHW-OAX events is lower in the Equatorial Pacific, where increased temperatures during El Nino directly give rise to lowering pH (due to changes in the carbonate chemistry), but at the same time the reduction in CO_2_ solubility, together with the reduction in the upwelling of CO_2_-rich waters results in the net CO_2_ outgassing and pH increase (Burger et al. 2022). Overall, the combination of underlying trends in ocean warming, acidification, and deoxygenation (Gruber et al. 2021) and large‐scale climate modes can make compound extremes more likely, or prolonged or more intense with devastating effects on marine ecosystems. The *Blob*, a major MHW observed in the northeast Pacific during 2013–2015, stands as an example, where regional circulation and ENSO-driven warming led to a persistent MHW (Di Lorenzo nd Mantua 2016), which occurred along with anomalously low oxygen and high [H^+^] concentrations generating a triple compound extreme (Gruber et al. 2021).

All these extremes, their combinations and linkages to forced and internal climate variability, are likely to continue to pose a risk to the ocean systems in the future decades even under the more optimistic GHG mitigation scenarios, because their drivers are expected to persist and amplify (Gruber et al. 2021; Holbrook et al. 2019).

Coastal regions are particularly vulnerable to compound extremes because, in addition to anthropogenic warming, other natural or anthropogenic pressures may affect them (e.g., dust and other aerosol deposition, pollution from agricultural practices and from sewage treatments, etc.). Trawling can impose huge pressures to coastal and benthic ocean pH (Atwood et al. 2023). Coastal extremes can also lead to algal blooms that die and decay, consuming oxygen leading to deoxygenation spikes. Globally, climate warming both exacerbates the problems from eutrophication and reduces the introduction of oxygen to the interior of the ocean.

However, unlike extremes on land, our knowledge about compound extremes in the ocean is relatively limited, especially how they manifest themselves, propagate, and interact at depth, and this is due to lack of relevant observations. It is therefore far less understood how marine extremes might lead to tipping points, although the potential for such interactions might be just as large as over land.

### Combination of Extremes in the Ocean and Links to Tipping Elements

Different combinations of extremes, whether preconditioned or multivariate, spatially or temporally compounding, have distinct characteristics, such as temporal and spatial scales of occurrence, regional and/or seasonal development and thus may interact differently with tipping elements. Below we outline some of the most common compound extremes and their characteristics while their specific impacts are addressed in Sect. 3.4.

#### Marine Heatwaves and High Ocean Acidity Extremes (MHW-OAX)

Compound events of marine heatwaves and high ocean acidity extremes (MHW-OAX) are just starting to receive more attention (Burger et al. 2022). Based on remotely-sensed observations, surface MHW-OAX have been found to occur most likely in the subtropics and less likely in the equatorial Pacific and high latitudes, largely driven by long-term warming and acidification trends (Burger et al. 2022). The Arctic Ocean specifically represents an area where extreme warming due to Arctic amplification and CO_2_ uptake lead to faster acidification changes that greatly impact ecosystem diversity and production (Jewett and Romanou 2017). However, MHW-OAX events are hard to fully characterize observationally, in terms of duration, intensity, areal coverage, and frequency, because there are no synoptic, consistent, continuous, daily and adequately long records of ocean acidification as there are for SST. Particularly challenging is the detection of extremes below the surface due to the lack of subsurface observations.

**Fig. 7:**
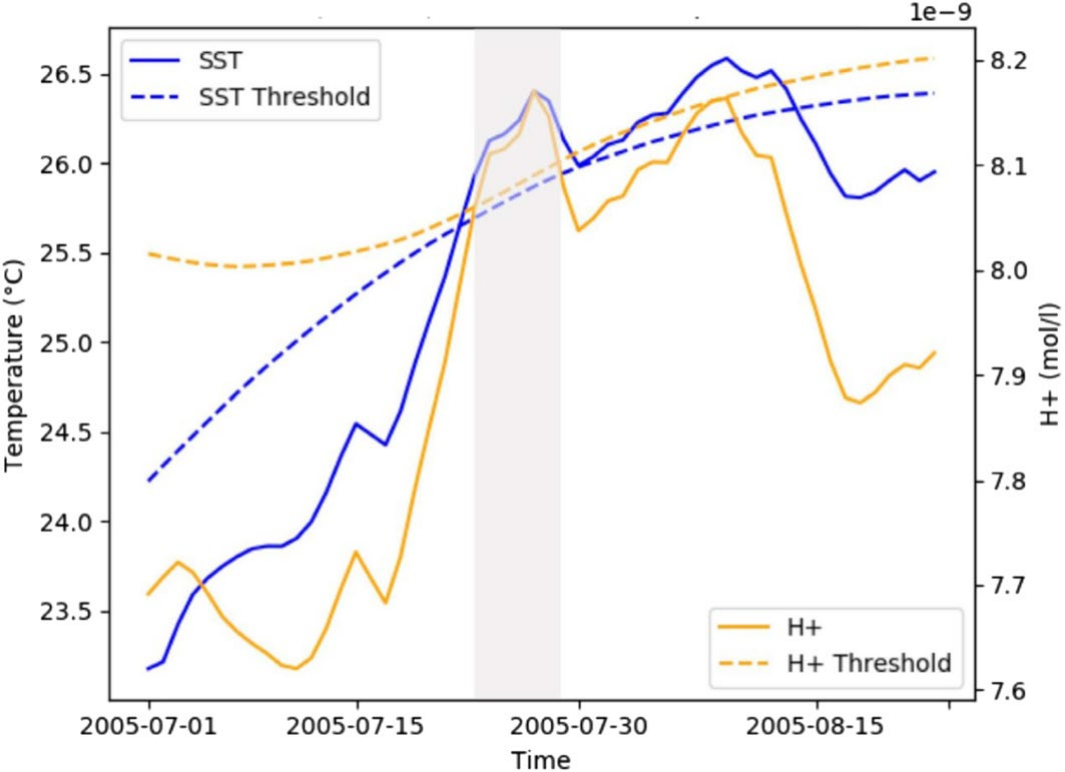
NASA-GISS modelE historical simulations: an example of a compound MHW (left axis) and OAX (right axis) event in the North Atlantic (highlighted in gray). Thresholds were computed using the 90-th percentile above a present-day baseline

On the other hand, climate models allow detection and analysis of MHW-OAX events at the surface and at depth, from high temporal frequency output, an example shown in Fig. 7 using the NASA-GISS climate model. Burger et al. (2022) in a similar model study showed that under 2 °C warming and associated acidification trends, surface MHW-OAX compound extremes, when computed relative to a fixed preindustrial baseline will last about 265 days per year, in contrast to just 12 days in preindustrial times.

While most studies focus on open ocean MHWs, nearshore extremes are more important for changes in the biogeochemistry and ecology of coastal waters which sustain not just commercial and recreational activities for nearby communities but also fisheries and aquacultures and therefore the food security and economy for large parts of the population. Nevertheless, it is understood that marine heat extremes near the coast, in enclosed bays and harbors, follow more closely the atmospheric variability than their open ocean counterparts (e.g., Cook et al 2022a, b and references therein). Similarly, coastal acidification has much larger variability than in the open ocean and therefore potentially stronger extremes; and is relevant for coastal ecosystems, including their tipping. Freshwater inputs there change seawater chemistry and make it more susceptible to acidification, by changing salinity, dissolved inorganic carbon, total alkalinity, dissolved and particulate organic carbon, and nutrients from riverine, estuarine and glacial melt sources. While these processes have persisted historically, climate-induced increases in glacial melt and high-intensity precipitation events can yield larger freshwater inputs than have occurred in the past. Nutrient runoff can increase coastal acidification by promoting phytoplankton blooms, which, when they die, fuel the bacterial respiration of CO_2_. These bacterial blooms can result in extreme acidity events whose intensity depends on local hydrographic conditions, including water column stratification and residence time. Long-term changes in nutrient loading, precipitation, and/or ice melt may also impart long-term changes in the magnitude of coastal acidification. Some high-latitude surface and near-surface waters already experience such corrosive conditions (Sutton et al. 2016; Bates and Mathis 2009; Qi et al. 2017).

This type of compound extreme happens near the coast and near the surface and thus may affect shallow corals and other ecosystems that cannot easily adapt to temperature and acidity changes, and at the same time affect aquacultures and fisheries. Because acidification rises quickly in coastal areas it might be the leading compound extreme for most of the ocean but more nearshore observations will be needed to understand the relationship between MWH-OAX and ecosystem tipping.

#### Marine Heatwaves and Ocean Deoxygenation Extremes (MHW-LOX)

Warming decreases the oceanic uptake of oxygen from the atmosphere, reduces ventilation of the ocean interior, and also increases the metabolic rate of marine organisms, and hence, their oxygen demand (Deutsch et al. 2015). Quantifying the combined indices of warming and deoxygenation, is done for example via the “metabolic index”, which represents the relationship between oxygen supply and demand at a standard metabolic rate (Deutsch et al. 2020) and the “aerobic growth index”, which uses growth theory, metabolic theory and biogeography to calculate a theoretical oxygen supply to demand ratio (Clarke et al. 2021).The long-term expansion of low-oxygen waters (Stramma et al. 2008; 2012) and increased metabolic oxygen demand have been found to result in habitat loss of heterotrophic organisms (Breitburg et al. 2018) and reduction of biodiversity, which may culminate in a mass extinction similar to those in Earth’s past (Penn and Deutsch 2022).

As the background temperatures and O_2_ levels change, O_2_-dependent biological processes become extremely susceptible to short-term MHW-LOX compound extremes (Köhn et al. 2022), and may lead to ecosystem tipping when the exposure to extreme conditions go beyond the realized niche limits (Trisos et al. 2020; Harris et al. 2018). Extinction risk is greatest where decadal climate anomalies are strong, or species are living close to their ecophysiological thresholds. Currently, the detection of open ocean LOX is hindered by the lack of O_2_ observational data with sufficient spatial and temporal coverage (Grégoire et al. 2021), and is generally carried out in model-data based studies (Gruber et al. 2021; Köhn et al. 2022). The combined effect of warming and deoxygenation on the metabolism and survival of tropical sea urchins and their exposure to compound extreme conditions occurring in their coral reef habitats has been shown to severely affect their survival (Lucey et al. 2022). While these species are able to withstand the extremes of heat or low oxygen separately, their combined effect leads to severe depletion of the population (Lucey et al. 2022). Also, the loss of coral reefs (Altieri et al. 2021) or fauna over seamounts (Ross et al., 2020) has also been attributed to deoxygenation as a cofactor.

#### Marine Heatwaves and Low Chlorophyll or Low Primary Production Extremes (MHW-LChlX, MHW-NPPX)

MHWs have been shown to lead to reductions in chlorophyll concentrations in the tropics and mid-latitudes and increases in high latitudes (Noh et al. 2022). However, coincident MHW and extremely low chlorophyll (LChlX, Le Grix et al. 2021) hotspots were found in the equatorial Pacific (Hughes et al. 2018), along the boundaries of the subtropical gyres, and in the Arabian Sea and the western Pacific Ocean (Chen et al. 2023).

The Blob was also associated with large negative anomalies in net primary production (NPP; Whitney et al., 2015; Mogen et al. 2022). Le Grix et al. 2023 assessed the characteristics of MHW-NPPX extremes in models and observations and showed that there are hotspots of frequent and long-lasting (> 10 days/yr) events in the equatorial Pacific and in the subtropical Indian Ocean, mostly associated with enhanced nutrient limitation on phytoplankton growth. In the northern high latitudes, they found much shorter events (< 3 days/year) due to enhanced light limitation, whereas large uncertainty between observational NPP products complicated the characterization of events in the Southern Ocean. Such extremes in low ocean productivity can be detrimental to upper trophic chains, e.g., pelagic fish species (Le Grix et al. 2022).

#### Other Marine Compound Extremes

Low frequency climate variability and air-sea interactions can spawn other combinations of extremes. MHWs in the central tropical Pacific have been observed to co-occur with extreme minima in sea surface salinity during intense precipitation events associated with El Nino (Liu et al. 2023a, b). However, low salinities have been associated with better adaptation to higher levels of acidity, especially in coastal regions (Xu et al. 2022; Li et al. 2022; Röthig et al. 2023) which might alleviate pressures from acidification or acidity extremes.

The co-occurrence of hypoxia and low pH, OAX-LOX, is also likely to occur particularly in shallow habitats, as microbial respiration consumes oxygen and produces CO_2_, with detrimental effects for coral survival and contributing, together with temperature anomalies, to seagrass mass mortality events (Altieri et al. 2021). Triple compound events, e.g., MHW-OAX-LOX, are also known to occur. In fact, the MHW known as the *Blob* has been associated with anomalously low oxygen, low pH, (Gruber et al. 2021), resulting in large negative anomalies in phytoplankton NPP (LeGrix et al. 2021; Morgen et al. 2022), as well as shifts in species distribution (Cavole et al. 2016; Cheung and Frölicher 2020) and severe consequences for marine life (Jones et al. 2018; Piatt et al. 2020).

These combinations of extremes are threatening for corals and other ecosystems as well as societal tipping points (collapse of fisheries etc.). For more details see Sect. 3.4.

Furthermore, SROCC (2019) has identified multiple cyclones as a kind of compound extreme, even though they happen sequentially, with potentially severe impacts on land and society. In that sense, sequences of MHW and other extreme events may also be identified as compound extremes. Such prolonged (or sequential) MHWs have been linked to the dynamics of the Pacific Decadal Oscillation (PDO) and the North Pacific Gyre Oscillation (NPGO) which are the two dominant modes of winter sea surface temperature variability in the North Pacific (Joh and DiLorenzo 2017).

#### Cross-Boundary, Atmosphere–Ocean and Land–Ocean Compound Extremes

Other compound extremes that affect the open-ocean and especially the coastal marine environment might occur over land or in the atmosphere. Examples include series of tropical cyclones in the Atlantic or the Pacific (Yaddanapudi et al. 2022; Swiss Re 2020) or storm surge-precipitation (Wahl et al. 2015; Wu et al. 2018), sea-level rise—storm surge—fluvial flooding (Ganguli and Merz, 2019a, 2019b; Moftakhari et al. 2017), and wind-precipitation events at a regional scale (Huang et al. 2021; Owen et al. 2021).

The *Blob* in the northeast Pacific was also concurrent with a record-breaking drought in California (Griffin and Anchukaitis 2014) that devastated the ecosystem there (see Sect. 3.4 below). Shi et al. (2021) identified similar events in the historical record that took place once in 1917–1918 and once in 1959–1960 but were not as extensive as the 2013–2015 event. Using CMIP6 models they showed that climate change will exacerbate such extremes by 2100.

Cross-boundary compound extremes include also compound flooding events due to extreme sea level rise and storm surge (Hoeke et al. 2013) or compound extreme river discharge and storm surge (Heinrich et al. 2022; Khanal et al. 2019; Couasnon et al. 2020). MHW coupled with extreme sea levels were found to be caused by greenhouse gas induced warming as well as wind decadal climate variability, associated with negative Indian Ocean Dipole (IOD) and a co-occurring La Nina that weakened the coastal upwelling (Han et al. 2022). Compound MHW with increased wind stress that drove enhanced southward transport in the Eastern Australia current may have led to the 2015–2016 Tasman Sea strong MHW that lasted for 251 days (Oliver et al. 2017). Cyclones, fronts and thunderstorms are associated with compound extremes in precipitation and winds, or gustiness and extreme ocean waves (Messmer and Simmonds 2021; Catto and Dowdy 2021). These are mostly found in North America, Japan, the Mediterranean, Australia and regions with high tropical cyclone occurrence. Cyclones associated with compound extremes were found to last longer and wind extremes were exacerbated in the presence of precipitation extremes. Compound low Arctic sea ice and anomalous SSTs can lead to anomalous jet stream meanderings that may have been responsible for the extreme cold spells over North America in 2015 (Bellprat et al., 2016).

In summary, the distinction between land and ocean extremes, single or compound, misses some interlinked extremes, whose consequences can ripple through the Earth system and contribute to long-term change.

### Tipping Points Potentially Triggered by Marine Extremes

#### Tipping in the Physical System

Compound marine or cross-boundary extremes can have impacts on the regional climate. For example, the Tasman Sea MHW of 2015–2016 led to drought followed by intense rainfall which caused severe bushfires and flooding in northeast Tasmania. Similarly in 2017 in coastal Peru, strong shallow ocean warming that reached 10 °C caused heavy rainfall and flooding (Garreaud 2018). MHWs have also been linked to decreases in sea ice (Laufkötter et al. 2020 and references therein) and could trigger local weather effects that modulate internal variability and therefore may affect local or even regional climate. Sea ice extremes may lead to tipping of the AMOC with impacts to global climate (Fig. 5A, Romanou et al 2023). MHWs have also been linked to changes in the Indian Summer monsoon (Saranya et al. 2022).

While there are few examples of extremes leading to changes in the physical climate, such impacts might be more ubiquitous than presently known and more possible occurrences should be explored in improved and expanded (in areal coverage and spatiotemporal resolution) observational records and in models.

#### Tipping of Ecosystems, Ecosystem Services and the Blue Economy

Some impacts of isolated extremes on ecosystems and ecosystem services have been well documented (e.g., Smith et al. 2023 for MHWs) and show that extremes can have more significant and more immediate consequences compared to the impacts of the long-term trends due to climate change. Additionally, the ensuing environmental degradation can lead to decline in the quality of food, water and other resources, such as wood, oil and medicinal plants, cultural and commercial importance of water resources (Techera and Winter 2019) and eventually the blue economy, as defined by World Bank (https://www.worldbank.org/en/programs/problue/overview): “the sustainable use of marine resources for economic growth, improved livelihoods, and jobs while preserving the health of ocean ecosystem”. Therefore, extremes may be able to lead to tipping of global or regional elements before climate change does.

MHWs cause biomass decrease and shifts in biogeography of fish stocks that are at least four times faster and bigger in magnitude than the effects of decadal scale mean changes throughout the twenty-first century (Cheung and Frölicher 2020). They may also bring on increased mortality of sea birds, salmon, marine mammals, low ocean primary productivity, harmful algal blooms, coral bleaching, defoliation of sea grass and decrease in kelp biomass, changes in biodiversity patterns of sessile invertebrates and demersal fish (although this is debated, Fredston et al. 2023), shifts in community structure and poleward shifts in tropical fish communities, closure of fisheries, locally large carbon outgassing events (Arias-Ortiz 2018). MHWs affect physiological adaptation and cause population and ecosystem changes (Smale et al. 2019; Almeida et al 2024) that are often dependent on the life stage (Dahlke et al. 2020), the organisms' physiology, on population and ecological dynamics and on the co-occurrence with other stressors. On short-term scales, the impact of extremes depends on the plasticity of the organisms (i.e., the physiological mechanisms that permit individual organisms to acclimatize to cope with environmental stress). Over longer timescales, there may be irreversible changes in ecosystem structure and composition (Smale et al. 2019; Straub et al. 2019), as for example, in communities of seagrass (Thomson et al. 2015), coral reefs (Hughes et al. 2019, 2020; Darling et al. 2013); micronekton (Brodeur et al. 2019) and kelp forests (Wernberg et al. 2021; Filbee-Dexter 2020; Michaud et al. 2022; Bell et al. 2023; Tait et al. 2021; Arafeh-Dalmau et al. 2019). Several studies have pointed out that it is often more detrimental for organisms, e.g., corals, when multiple MHWs act simultaneously (Smith et al. 2019; Anthony et al. 2008).

On the other hand, both long-term ocean acidification and extremes of pH (especially in estuaries and coastal waters) reduce the rate of calcification, resulting in decreased ability of marine organisms, such as corals, plankton, and shellfish, to build their shells and skeletal structures, as well as decreased reproduction, disease and death. This can result in ecosystem shifts and loss of the structural complexity and biodiversity, exacerbate existing physiological stresses and can affect the growth and survival, respiration, and reproduction of many non-calcifying species, potentially impacting photosynthesis and primary production from benthic species and corals up to fish (Gao et al. 2019; Tassone et al 2022).

Ocean acidity and warming compound extremes can have synergistic or antagonistic effects although some adaptation might take place. While warming might increase some behavioral responses (such as swimming speed), ocean acidification tends to restrict them (Baag and Mandal 2022). Little is known about impacts on the reproductive ability of many organisms, like copepods, reef fishes, squid, garfish, clams, sea urchins, oysters, the Atlantic cod, the Pacific herring (Baag and Mandal 2022 and references therein). Shell, skeletal and muscle deformities as well as other physiological changes are known to affect swimming behavior and feeding rate. Pathogen growth, disease spreading and parasite transmission is changing under climate change and thermal and acidification stress, as shown for lobsters, gastropods, Atlantic Halibut, and other species (Doney et al 2020 and references therein).

Such impacts will affect both fisheries but also aquaculture with enormous consequences and socio-economic implications on commercially important fisheries and threaten food security, livelihood, and employment to local but also regional communities.

Corals are particularly vulnerable species to MHW-OAX compound extremes. Over the past decades large-scale coral bleaching events in the tropics have become common (Baker et al. 2008; Spalding and Brown 2015; IPCC, Bindoff et al. 2019; Fordyce et al. 2019; Klein et al. 2022) and are linked to the occurrence of MHW and compound events, particularly. The decline in coral production affects the global ecosystem, with biodiversity reduction and collapse of ecosystem services such as food production and coastal protection and socioeconomic impacts (Brander et al. 2012; Robinson et al 2019).

Combined MHW and LOX events will lead to decrease of habitat viability globally (Kim et al. 2023), but will affect different species differently, and may even lead to extinction (Penn and Deutsch 2022). For example, even short duration (~ 1 day) events can be catastrophic for sea urchins (Lucey et al. 2022). Under deoxygenation stresses, most species will lose about 5% of their habitat at 2 °C warming levels (above preindustrial temperatures), some species will lose double or triple that. Deoxygenation is particularly important for mesopelagic and demersal habitats. However large uncertainties exist in the vulnerability and species-specific biophysical thresholds response (Moree et al. 2023).

Extreme changes in ocean stratification and sea ice extent (as seen in Blanchard-Wrigglesworth et al. 2021; Holland et al. 2008) in the subpolar gyre (SPG) can lead to SPG mixing and AMOC collapse which in turn will cause reduction or cessation of deep-water formation and eventually primary production collapse (Schmittner 2005) and deoxygenation (Schmittner et al. 2007; Schmittner and Galbraith 2008).

Prominent compound extreme events such as the *Blob* have had severe impacts on marine life (Cavole et al., 2016), including extreme mortality and reproductive failure of sea birds across the north-west coast of the US (Jones et al. 2018; Piatt et al. 2020), persistent harmful algal blooms, mass stranding of whales in the western Gulf of Alaska and of sea lions in California, and replacements with warm-water species (Cavole et al. 2016; Cheung and Frölicher 2020). Additionally, there were devastating economic and ecological consequences in the terrestrial realm as well (e.g., Lund et al. 2018; Peterson et al. 2017; Santora et al. 2020). Drought led to groundwater overdraft, land subsidence, loss of domestic water supply in the Central Valley (Bee 2015), and multi-billion-dollar agricultural losses from reduced farmland productivity and additional groundwater pumping costs (Howitt et al. 2014, 2015; Medellín-Azuara et al. 2016). This severe and extended drought killed more than 100 million trees, exacerbated the devastating 2015 fire season, and remains a fuel hazard that continues to threaten public safety presently. The economic loss was enormous. For example, about $170 million losses were due to the closure of the Dungeness crab fishery alone (McCabe et al. 2016) while ∼$120 million was allocated for drought emergency ecosystem support from state and federal sources (Hanak et al. 2015).

Gruber et al. (2021) point out that deep extensions of compound extremes below the ocean surface will lead to additional habitat compression, and we need to better understand subsurface signatures of the extremes to improve preparedness and reduce disaster risk, including that of tipping points linked to compound ocean extremes discussed above.

In conclusion, marine extremes could have a strong role in short-term but also long-term shifts in the Earth System, and perhaps approaching or exceeding tipping points with potentially serious consequences for marine ecosystem services.

## Palaeoclimatic Evidence of Link between Tipping Points and Extreme Events

Risk of tipping points is supported by palaeoclimatic evidence, and mechanistic links suggest a role for extremes, although particularly weather extremes have generally not been directly evidenced in palaeoclimatic literature. In contrast, climate events (as defined above) have seasonal signatures and thus can be resolved in some archives.

Drought atlases over the common era (Cook et al. 2022a, b) can begin to shed light on the largest possible seasonal events, and their links with tipping points such as vegetation shifts, and impacts on societies. Megadroughts over the Common Era, with multi-decadal to longer and greater spatial extent than the twentieth century, are documented in records, such as historical documents, tree rings, archaeological remains, lake sediment, and geomorphic data, for the central U.S. (Woodhouse and Overpeck 1998) and southwestern North America (Williams et al. 2020). Fire data are also available across interglacial and recent periods and may help to identify possible links between long-term change and fire. Paleoecological records provide a link of climate, vegetation, and past fires. Frequent fires in boreal Siberia during the late Holocene led to a shift in vegetation to fire-adapted forests (Feurdean et al. 2020). There are also reconstructions of severe storm activity resulting in flooding. A multi-proxy record from central Texas indicated an abrupt and rapid increase in mesoscale convective system activity and the establishment of overall wetter conditions at ~ 14,500 years ago, albeit associated with orbital forcing and deglacial conditions (Sun et al. 2021). Variability in regional intense hurricane activity over the Common era has been detected in lacustrine sediments (Brandon et al. 2013; Rodysil et al. 2020).

Over ocean, corals can provide annual to seasonal resolution and thus resolve climate timescale of extreme events, while other proxies provide less temporal resolution. On the other hand, studies such as Hillyer et al. (2017) and Roach et al. (2021) employ high-resolution sampling of coral skeletons to detect metabolomic changes associated with thermal stress and bleaching, which may help capture signatures of short-term events like MHWs in historical coral records; but also importantly directly document impacts of extremes. This is also the case for tree ring data which record stress on vegetation, something that could be exploited for characterizing impact of extreme events. Advancements in high-resolution sampling, multiproxy approaches, and climate modeling may provide some pathways to infer the occurrence and impacts of these events, although short-term MHWs may remain elusive. For the discussion following below it is an open question if extremes played a role in rapid transitions and one that would be helpful to explore, either from high resolution data or model simulations in order to better enable early warning.

Paleoclimate records also suggest a strongly nonlinear, complex climate system, and reveal instances where there have been abrupt changes in the state or modes of variability of components of the climate system (Rahmstorf 2001; Lockwood 2001; Alley et al. 2003; Rial et al. 2004). These previous shifts point to potential tipping elements of the climate system, are not able to provide, and climate modeling of these transitions as well as analysis of high resolution archives may elucidate the drivers behind these shifts. There are a few candidate periods for doing so:

During the African humid period (AHP) approximately 14,700 to 6,000 years ago, northern Africa experienced a wetter environment in which the hyperarid Sahara was transformed into a mesic landscape, with a northward shift of tropical vegetation by approximately 6–9° latitude compared to the present-day location, large permanent lakes, and extensive river drainage networks (Drake and Bristow 2006; Lézine et al. 2011; Hély et al. 2014; Tierney et al. 2017, Pausata et al. 2020). This favorable climate was influenced by a slightly increased tilt of the Earth's spin axis and perihelion in July, resulting in stronger insolation in the Northern Hemisphere during the summer, which in turn strengthened the West African summer Monsoon. Moreover, increasing greenhouse gas concentrations at the start of the AHP (~ 14,700 years ago) could have further enhanced precipitation in northern Africa (Otto-Bliesner et al., Science 2014). Climate records into and out of the AHP suggest that the transitions were relatively rapid in some regions (deMenocal et al. 2000; Adkins et al. 2006; McGee et al. 2013; Tierney et al. 2017), while they were more synchronized with orbital forcing in other regions (Niedermeyer et al. 2010). New transient climate simulations show spatially heterogeneous vegetation dynamics in northern Africa, with the fastest increase in simulated desert fraction—up to 7 times faster than orbital forcing—occurring around 20°N in the western part and around 14°N in the central and eastern part (Dallmeyer et al. 2020), while the transition was more gradual in other regions in synch with orbital forcing (Shanahan et al. 2015; see also Kutzbach and Guetter 1986). In conclusion, the AHP transition of the Sahara was regionally rapid, suggesting the crossing of a tipping point with respect to changes in the driving forcing. However, when viewed within the context of human lifetimes and local ecosystems, this transition unfolded gradually, although it may have come in the shape of a changing extremes regime, similar to how we experience climate change today. The AHP transition, whether rapid or gradual, brought about enduring and significant transformations. These changes acted as a pivotal moment in human civilizations, giving rise to new cultures along the Nile.

The paleoclimatic record can also inform on the impact of strong high latitude warming on vegetation and the polar ice sheets. A strong northward shift of the boreal vegetation over Canada and Eurasia, combined with forest loss in Southern regions, has been reconstructed in past warm periods, such as the Eemian, although with significant uncertainty on the extent of this retreat, and its spatial homogeneity (e.g., Otto-Bliesner et al. 2020; Thomas et al. 2020 and references within). Presently, changes in the boreal forest toward deciduous vegetation are small (Massey et al. 2023) with difficulty in assessing its resilience using EO data (Smith and Boers 2023). Genetic evidence from a circum-Antarctic octopus indicates that the West Antarctic Ice Sheet (WAIS) WAIS collapsed completely during the Last Interglacial period, when global average temperature was like that of today (Lau et al. 2023). Identifying the precise tipping point of the WAIS during the Last Interglacial, the triggers, and its connection to extreme events are still outstanding questions.

## Observing, Understanding and Predicting Risk of Extreme Event Related Tipping

### Earth Observation Monitoring Capabilities

When we approach a tipping point, we expect that the climate variability will increase (Scheffer et al. 2009), thus also making some extreme events more likely. A careful monitoring of extreme events could thus help identify situations in which we are close to a critical threshold (see companion paper by Bathiany et al. 2024). When applying this method, it is important to fully consider uncertainty in observations, where changes in the observing system or data availability can also lead to changes in variance; and to fully consider climate variability and decadal drivers of it. Further evidence on tipping risk includes monitoring the ecological or physical health or degradation of a system at risk of tipping.

Earth observations can capture *extreme weather and climate events*. Land surface temperature is recorded by MODIS (Moderate Resolution Imaging Spectroradiometer) on NASA’s Terra and Aqua satellites, and by SLSTR (Sea and Land Surface Temperature Radiometer) on Copernicus Sentinel-3 (Nickeson 2023; Yu et al. 2022a, b.; and Good et al. 2022). These can be combined with reanalysis products that date back to the nineteenth century (Slivinski et al. 2019). Reanalyses also capture past extreme heat reasonably well (e.g., Cowan et al. 2020a), and together with satellite data enable identifying extremes in remote or under-recorded regions (e.g. Engdaw et al. 2023). There are offsets between data sources, and retrieval biases, yet identified extreme events show good agreement between reanalysis and satellite data (e.g., Hegedűs et al. 2024). However, Observational products like OISSTv2 and other higher level satellite products have coarse resolution near the coast and thus fail to reproduce observed MHWs in the estuaries and the land–ocean continuum (Cook et al. 2022a, b). This highlights the need for long-term in situ measurements as well as methods to improve the algorithms that produce the satellite effective estimates near the coast.

For precipitation, EO data are useful particularly for remote regions and over the ocean. Blended satellite/in situ records (GPCP; Adler et al. 2018) record global precipitation, although high latitude precipitation retrieval is affected by ice and snow. Precipitation deficit is one possible definition of drought, although accounting for evaporation in P-E or the Palmer drought severity index captures stress on vegetation much better. The latter also shows substantial increase (IPCC 2021; Seneviratne et al. 2021) partly due to added evaporation by global warming.

Multiple satellite-based datasets are available for *monitoring vegetation and fire* (e.g., Jiao et al. 2021), over multiple decades, as AVHRR, Landsat and MODIS, or more recently Sentinel-2 and Sentinel-3 (Lastovicka et al. 2020; Li et al. 2024). Satellite-based information about vegetation condition can be derived from vegetation indices such as the normalized difference vegetation index (NDVI Tucker 1979), a proxy for leaf area and productivity; normalized difference water index (NDWI Gao 1996), a proxy for drought stress, and passive microwave vegetation optical depth (VOD, Liu et al., 2011), which responds to combined biomass and vegetation water content. NDVI may be problematic over dense vegetation due to saturation effects (Smith et al. 2022). Solar-induced chlorophyll fluorescence is a more recently available satellite-based proxy for photosynthesis (Sun et al. 2023), although it's use to monitor extreme event impacts is still under debate (Martini et al. 2022).

There are also remotely sensed data for active fires and fire radiative power (Xu and Wooster 2023) from Sentinel-3, for normalized burn area (Chuvieco et al. 2018), burn area and fire intensity (Giglio et al. 2016). Passive optical satellite retrievals are affected by cloud cover, which introduces a bias compared to surface air temperatures (e.g. Nickeson 2023). All of these are able, with careful analysis, to evaluate to what extent vegetation recovers and with what delay, and how severe changes are. This applies to all extreme events affecting vegetation, from heat, drought and fire to severe storms, and other vegetation disturbance. For an in-depth discussion of remote-sensing based assessments of vegetation resilience, see Bathiany et al. 2024.

*MHW* are identified primarily using SST from satellite thermal infrared measurements (e.g. Advanced Very High Resolution Radiometer (AVHRR) on NOAA Polar-orbiting Operational Environmental Satellites (POES), Along-Track Scanning Radiometer (ATSR) aboard the European Remote Sensing Satellite (ERS-2), the Geostationary Operational Environmental Satellite (GOES) Imager, and Moderate Resolution Imaging Spectroradiometer (MODIS) aboard NASA Earth Observing System (EOS) Terra and Aqua satellites, VIIRS on Suomi NPP) or passive microwave measurements (e.g. the Scanning Multichannel Microwave Radiometer (SMMR) carried on Nimbus-7 and Seasat satellites, the Tropical Rainfall Measuring Mission (TRMM) Microwave Imager (TMI), and the Advanced Microwave Scanning Radiometer (AMSR) instrument on the NASA EOS Aqua satellite and on the Japanese Advanced Earth Observing Satellite (ADEOS II), Sea and Land Surface Temperature Radiometer (SLSTR) on Sentinel-3). Infrared radiation of the ocean comes from the top 10 microns of the surface while microwave radiation results from the topmost 1-mm layer. Infrared satellite sensors have better spatial resolution but are more susceptible to cloud contamination than microwave. Today in addition to satellite and shipboard measurements there are thousands of floats in the oceans measuring temperature and salinity and more recently key biogeochemical variables (e.g. the ARGO floats). These are used to validate satellite instruments in addition to sampling throughout the water column. The surface drifters from the NOAA Global Drifter Program (GDP) regularly provide about 60,000 night time SST measurements per month at a shallower depth of 0.2 m, while the Group for High Resolution Sea Surface Temperature (GHRSST) project provides all SST data sets in a common format that allows for easy accessibility across different computer platforms and operating systems and to make it useful to the modeling community also provides a full characterization of the errors associated with each pixel.

The use of satellite-based observations can provide global gap-free SST datasets at high spatial and temporal resolution, typically covering the period from 1979 to present (Yang et al. 2021). Hobday et al. (2016) was the first to define a set of quantitative globally valid metrics to detect and classify MHW events. Since that work, studies on MHWs have been increasing. There is a better understanding (Holbrook et al. 2019; Oliver et al. 2021), although still not complete, of the sensitivity of the MHW detection methods, and the physical processes that drive temperature changes in the mixed layer, leading to the build-up, persistence, and decay of MHWs, and how local internal variability (e.g., eddy instabilities) and large-scale climate variability possibly modulate the severity of these events locally (Collins et al. 2019; Frölicher and Laufkötter 2018; Jacox 2019). Yet, mechanisms leading to subsurface MHW are still poorly constrained. Furthermore, studies investigating the impacts of MHWs on atmospheric circulation and the rest of the physical system are very limited.

Aside from SST, satellites measure sea surface salinity (SSS) and sea level rise (SLR) through the recent advances with ESA’s SMOS and NASA’s Aquarius and SMAP missions—see Vinogradova et al. (2019) for salinity and the Gravity Recovery and Climate Experiment (GRACE) and Jason − 1 and − 3 missions for SLR.

Satellite-based estimates of chlorophyll and primary productivity come from the Moderate Resolution Imaging Spectroradiometer (MODIS) on NASA's Aqua satellite and ESA’s Ocean and Land Colour Instrument (OLCI) on Sentinel-3. However, we lack similar quality, synoptic information for oxygen and H+ concentrations in the ocean, as well as consistent and continuous monitoring of the subsurface (and surface) ocean.

For large scale and regional tipping elements we need consistent and continuous monitoring of the higher spatial and temporal scales to be able to better assess extent, frequency, duration and intensity as well as deep signatures of marine extremes (see Wood et al. 2024).

Emphasis should be placed on global (including high latitudes), high frequency measurements, from satellite missions, e.g. through the anticipated launch of the Plankton, Aerosol, Cloud and Ecosystem (PACE) mission, and the new mission to address submesoscale e.g. the Surface Water and Ocean Topography (SWOT), as well as suborbital platforms, such as planes, ships, moorings, transects, more bioArgo, but also instruments that can capture the development of extreme events e.g. gliders, AUVs through the Lagrangian rather than the Eulerian view of the flow (Gruber et al. 2021). Since the subsurface ocean but also the overlying state of the atmosphere are of critical importance to our understanding of how physical and biogeochemical extremes grow and interact and how they might lead to tipping points, Ocean Super Sites (Clayson et al. 2021) will provide high temporal monitoring to possibly capture early warning signals for tipping points (as described in Scheffer et al. 2009).

An important point that needs to be made abundantly clear (as shown and discussed in Burger et al 2022) is that the choice of baseline is critical in impact studies. Simply a historical baseline in the determination of an extreme event might be sufficient for corals (i.e. immovable species, even though it still does not take into account adaptation capabilities) but still it is not appropriate for assessing impacts on fish (i.e. species that can migrate and change habitat). Even in this case, however, loss of habitat and biodiversity are phenomena that can be illustrated using a constant baseline.

### Climate Modeling and Machine Learning Perspective

Understanding mechanistic connections between extreme events and long-term impacts on the Earth System can also draw on Earth System Models (ESMs), which are scoped to include not only the climate system but also its interaction with the global carbon cycle, terrestrial vegetation, and marine biogeochemical cycles. However, not all modeling groups perform ESM scenario simulations with all interactions switched on. For example, while several models used in AR6 chapter 5 included fire (Table 5.4, Canadell et al. 2021), only 2 models allowed for interactive vegetation and hence a response of vegetation to fire. The potential consequences of fire and drought are therefore likely underestimated, or not captured, in past generations of ESMs. It is also important to evaluate and constrain model responses with observations, as uncertainties are substantial (for example, due to inadequate model complexity; or model ability to capture diverse vegetation/climate systems). Comparisons, for example, between fire models and data indicate underestimated fire responses (e.g., Sanderson and Fisher 2020 for Australia). Major uncertainties exist around ignition and vegetation response (Teckentrup et al. 2019), and the few most complete fire models appear to underestimate the size of fires, which could lead to underestimation of the effect of large extremes and fire (Hantson et al. 2020). EO will provide a vital tool to evaluate ESMs with coupling switched on and determine if they realistically estimate risk from large extreme events and associated fire. Similar issues also arise for other tipping points, such as those associated with ice sheets. Ice sheet models still largely run offline rather than fully coupled and hence will not ‘see’ extreme warm events or warm ocean incursions, while also being unable to influence ocean dynamics with melt water (Schmidt et al. 2023; Roach et al. 2023). On the ocean side, not all CMIP6 models include fully prognostic and interactive dust or river runoff while subdaily output simulations typically do not cover the breadth of each model’s internal variability.

The move toward high resolution has the potential to improve the link between systems due to better resolving the scales required for coupling (e.g., Lee and Hohenegger 2024), although that is not always the case (Ridder et al. 2021). The high computation requirements also affect the potential to sample extreme events in shorter runs, and to fully account for irreversibility.

Ocean modeling, particularly Lagrangian back-trajectory analysis (Holbrook et al. 2020) and data-assimilation of the sparse in situ data, but also forward modeling (climate or process modeling) can provide invaluable insights on the onset, persistence, decay, spatial extent, depth, and intensity as well as future projections of the extremes, their drivers (Burger and Frölicher 2023), and their interactions with the tipping elements.

Very importantly, climate model skill on representing compound extremes needs to be analyzed using multivariate bias-assessment framework, and not singular and sequential individual parameter evaluation, in order to reliably depict the models’ capability in assessing risk due to compound extremes (Villalobos-Herrera et al 2021).

In that context, machine learning (ML) enables bridging scales between processes on very fine resolutions, such as ice sheet melting, and larger scale models and also in detecting links between extreme events and tipping. As changes in vegetation or biosphere are generally not mono-causal, AI causality methods can also be used to determine driving factors and responses and identify the most important mechanisms (e.g., Runge et al. 2019).

Furthermore, ML helps in feature detection (Gruber et al. 2021), including space–time coherent extreme events (e.g. Hegedus et al., 2024), identification of precursors, improved prediction (Holbrook et al. 2020), downscaling and bias correction that leads to better study of extremes and their impacts. However, ML limitations include the non-stationarity of the climate solutions, potential for unphysical outcomes, difficulties in uncertainty quantification, and importantly the limited sampling of extremes in observational data due to sparsity of data and short records. In other words, ML can only be as good as the best observational and model data we have, and needs to be accompanied by understanding of mechanisms. To improve predictability of extremes, as well as efficient detection and attribution of the compound extremes, we will need to overcome observational limitations which can be done via emulation of climate predictions at ultra-high resolution (1 km or less) (Molina et al. 2023).

Overall, the large uncertainty in the carbon cycle response to increased warming affects our understanding of the remaining carbon budget. As extremes and tipping points are likely important sources of this uncertainty, better monitoring and simulating of the cascading effect of extremes and their possible impact on tipping points is required to address this uncertainty, and the reversibility of changes associated with extreme events.

## Conclusions and Outlook

### Summary and Discussion

The importance of extremes, and especially compound extremes, in inducing tipping points is evident from the discussion of both marine and land systems. Additionally, if extremes are defined relative to preindustrial or even late historical conditions, they are expected to become near permanent in large parts of the world even under high mitigation scenarios of greenhouse gases (Batibeniz et al 2023). Efforts should be placed to address cross-system (land–ocean-ice-atmosphere) compounding extremes, e.g. extreme precipitation over land and/or river discharge or wind spells coupled to MHWs, etc. Furthermore, governance and economic markets may create links between global extremes and complicate (exacerbate or alleviate) physical and biogeochemical extremes (Raymond et al 2020). In this context, Earth observations could be of high value to monitoring extremes as well as many of these interactions and tipping points and are therefore a vital tool for early detection. Detection should make use of early warning systems, which could be based on remotely sensed extreme land and ocean surface temperatures, observations of vegetation or crop stress or dieback indicators etc. However, new machine learning and AI techniques may be more successful in identifying early warning systems. Climate prediction systems can provide early warning of atmospheric and ocean variability, which can enhance the probability of individual and compound extremes.

While we might have to reconsider the climate risk assessment framework and target plausibility and not only probability of extremes (Sillmann et al 2021), a potential roadmap for research at the interface between extreme events and tipping points would include the following elements:

- Foster exchanges and the development of a community working at the interface between extreme events and tipping points, to develop interdisciplinary research on the potential of triggering tipping points by extreme events. This should include both land and ocean communities, draw on improved process understanding, and integrate information from models and observations using statistical and ML techniques.
- Ensure detailed discussion and process oriented treatment of high risk events and tipping points in upcoming assessments, e.g. the next report by the Intergovernmental Panel on Climate Change.
- Encourage new, high frequency, spatially and temporally, in-situ observations near the coasts, estuaries and inlets, improve and expand satellite retrieval algorithms to obtain better satellite-based estimates of extremes near the coast.
- Ensure continuous and consistent measurements of key observables to maintain long enough records for climate trends, and also monitoring of high frequency variability to identify potential critical conditions and early warning signs of tipping points.
- Work towards fully interactive coupling of relevant processes in climate models and all their subcomponents, such as prognostic ice sheets/glaciers and their feedbacks with climate, links between ocean biogeochemistry and dynamics (e.g. effect of mesoscales and submesoscales), two-way feedbacks between land vegetation and climate, coupling between ecological and hydrometerological processes (e.g. Mahecha et al 2024), resolving processes in the land–ocean and the air-sea interfaces. EO data are essential to evaluate the model components individually and in their interactions.
- Explore the potential to use AI to bridge scales and, target in particular high spatial and temporal resolution processes.
- Employ a new methodology for attribution to anthropogenic climate change for compound vs single extreme events (e.g. Zscheischler and Lehner 2022).
- Integrate human factors beyond climate forcing (e.g. land management, deforestation, coastal pollution) to better capture the interaction of climate and other man-made pressures.
- Improve monitoring of extreme events and the recovery from them in space and time, in order to better understand current model limitations and biases, including in determining long-term (multi-decadal) change.

### Open Research Questions

In the following, we highlight some open high priority research questions that would be useful to address in the emerging research area of extremes-induced tipping points; and to better enable early warning and adaptation.

1. What types of extreme events are the most dangerous for triggering long-term change or tipping; what are their early warning signs and is there a possibility to take mitigative action that avoids tipping and also reduces the impact of extreme events on the carbon cycle, climate and society? On the other hand, do some extremes such as cold events (e.g. due to natural variability or volcanic eruptions) increase the resilience to tipping?
2. How realistic are ESMs in capturing the most extreme events and their full impact, and its recovery time, resilience, and reversibility?
3. What are the carbon implications of the most severe extreme events when all cascading effects and recovery timescales are fully considered?
4. What is the potential for socioeconomic tipping in response to severe extreme events, and what are the most important triggering mechanisms?
5. How can the links between extreme events and tipping points be better captured in tipping point and nonlinear response theory?
6. Lastly, in this review, we treated the land- and ocean-based extremes-driven tipping points in isolation, but there are also potential links between the ocean and land tipping points / extreme events, and those links need to be investigated: Examples are marine heatwaves leading to heavy rainfall over land, wildfire smoke plumes causing phytoplankton blooms in the ocean, snow/ice melting on land impacting ocean extremes/tipping points.
